# Supplementary material for: Reductive Amination for LC–MS Signal Enhancement and Confirmation of the Presence of Caribbean Ciguatoxin-1 in Fish
Source: Toxins (Basel). 2022 Jun 9;14(6):399. doi: 10.3390/toxins14060399 (PMC9245599; doi:10.3390/toxins14060399)
Supplement: Supplementary file 1 [file toxins-14-00399-s001.zip › toxins-1742505 SM_SU_EM.pdf]

Supplementary Materials: Reductive Amination for LC–MS Signal Enhancement and Confirmation of the Presence of Caribbean Ciguatoxin-1

Fedor Kryuchkov, Alison Robertson, Elizabeth M. Mudge, Christopher O. Miles, Soetkien Van Gothem and Silvio Uhlig

Table S1. A. Effect of time and temperature on the yield of C-CTX-1–GRT (**4**) in initial optimization trials using sodium cyanoborohydride as reducing agent. The reaction conditions shown in bold gave the highest yield of **4**.

Sample[1] volume (μL)	Acid[2] volume (μL)	GRT[3] volume (μL)	$NaBH_3CN$[4] volume (μL)	T (°C)	t (h)
40	20	20	20	60	0.5
40	20	20	20	60	1.5
**40**	**20**	**20**	**20**	**20**	**24**

Table S1. B. Effect of acid concentration on the yield of C-CTX-1–GRT (**4**) in initial optimization trials using sodium cyanoborohydride as reducing agent. The reaction time was 2 h, the reaction temperature was 60 °C. The reaction conditions shown in bold gave the highest yield of **4**.

Sample[1] volume (μL)	Acid[2] volume (μL)	GRT[3] volume (μL)	$NaBH_3CN$[4] volume (μL)	Volume MeOH added (μl)
40	0	20	20	40
40	5	20	20	35
**40**	**10**	**20**	**20**	**30**
40	20	20	20	20
40	40	20	20	0

Table S1. C. Effect of type of acid on the yield of C-CTX-1–GRT (**4**) in initial optimization trials using sodium cyanoborohydride as reducing agent. The reaction time was 1 h, the reaction temperature was 40 °C. The reaction conditions shown in bold gave the highest yield of **4**.

Sample[1] volume (μL)	Acid[5]	GRT[3] volume (μL)	$NaBH_3CN$[4] volume (μL)
40	AcOH	20	20
**40**	**FA**	**20**	**20**
50	TFA	20	20

Table S1. D. The yield of derivatization as a function of time and temperature for two-step GRT reductive amination of **1**, using sodium cyanoborohydride as reducing agent. The temperature and time values relate to a two-step derivatization. The reaction conditions shown in bold gave the highest yield of **4**.

Sample[1] volume (μL)	Acid type (1%) and volume (μL)	GRT[3] volume (μL)	$NaBH_3CN$[4] volume (μL)	T (°C)	t (h)
40	AcOH, 20	20	20	40; 40	1; 1
40	FA, 20	20	20	40; 40	1; 1
40	FA, 5	20	20	40; 40	20; 2
40	FA, 5	20	20	20; 40	20; 2
**40**	**FA, 20**	**20**	**20**	**20; 40**	**20; 2**

[1] Sample D [2] 10% formic acid [3] 1 mg/mL in methanol [4] 2 mg/mL in methanol
[5] 1%, 20 μL added; FA = formic acid

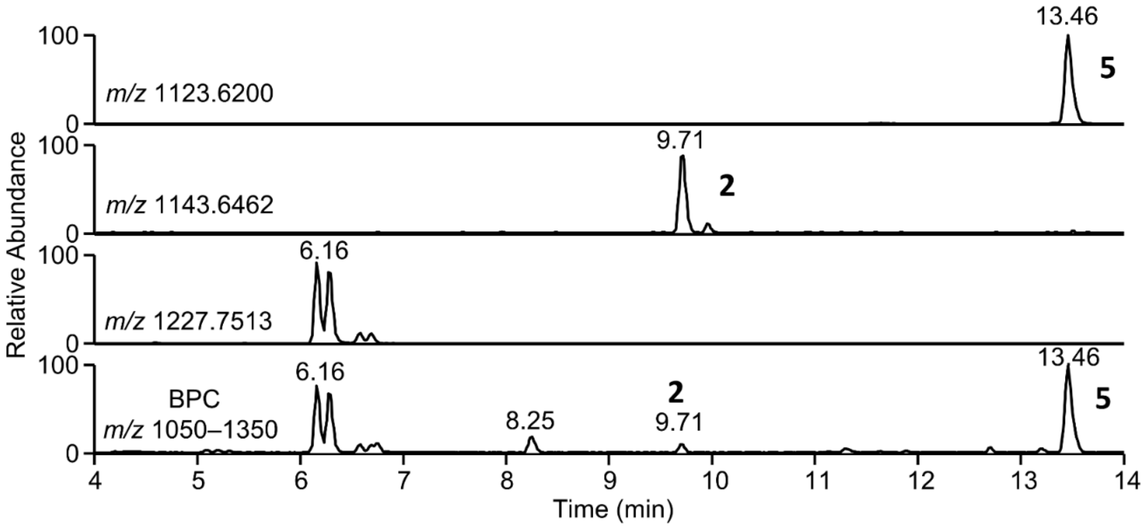

Figure S1. Chromatograms from LC–HRMS analysis (LC–HRMS method 1) of the reductive amination of **1** with AETMA (derivatization method 3) in a ciguatoxic sample. The desired products elute at 6.16 and 6.29 min and were observed at *m/z* 1227.7513. Chromatograms are plotted using a mass tolerance of ± 5 ppm. The base-peak chromatogram (BPC, bottom) gives an impression of relative base-peak abundances.

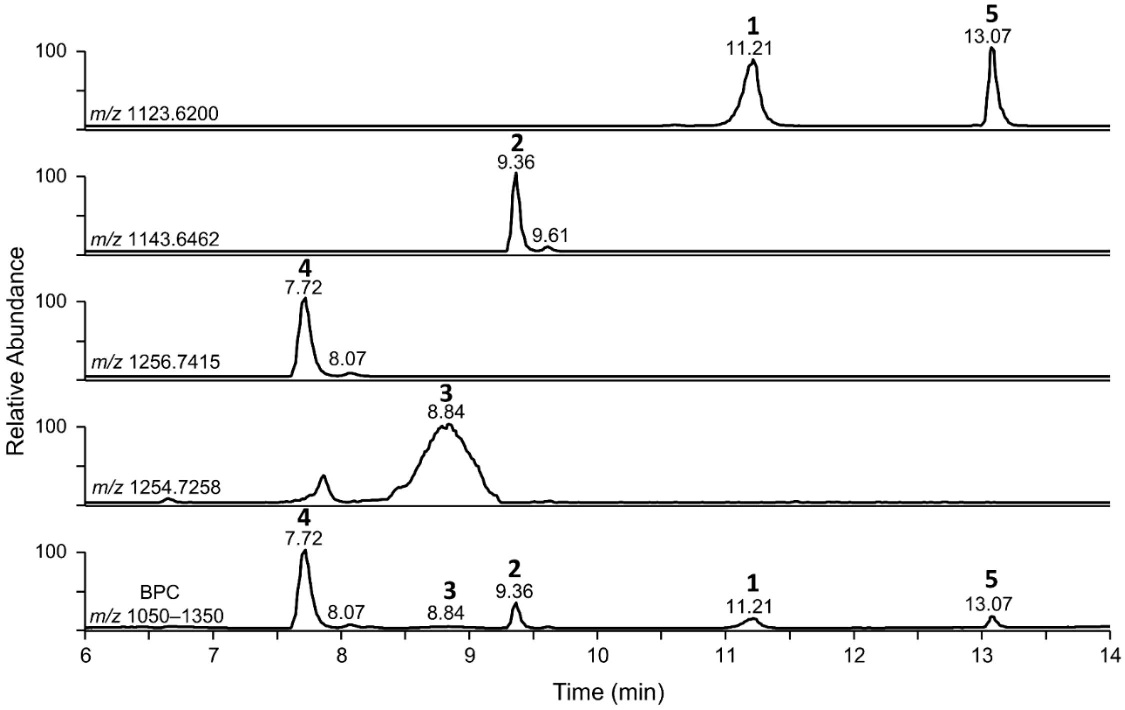

Figure S2. Chromatograms from LC–HRMS analysis (LC–HRMS method 1) of the reductive amination of **1** with GRT in a ciguatoxic sample under non-optimized conditions, showing formation of by-products. Chromatograms are plotted using a mass tolerance of ± 5 ppm. The base-peak chromatogram (BPC, bottom) gives an impression of relative base-peak abundances.

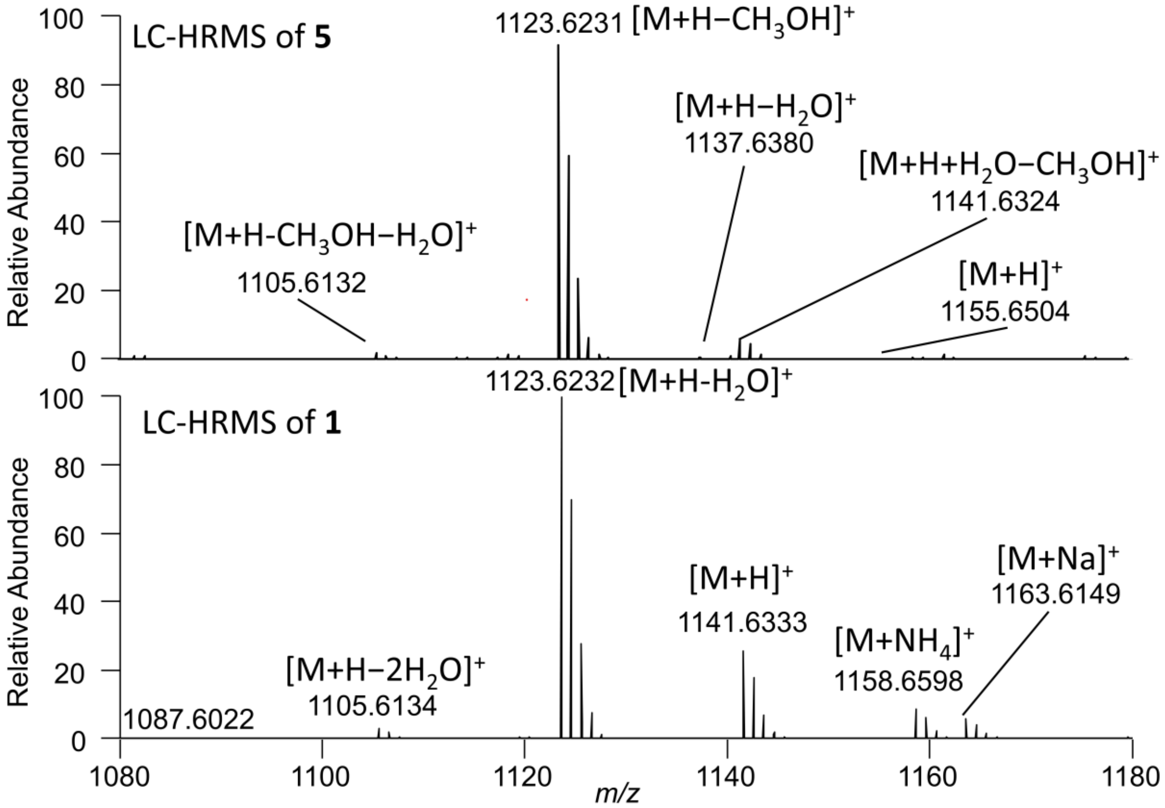

Figure S3. LC–HRMS spectra for C-CTX-1 56-methylketal (**5**, top) and C-CTX-1 (**1**, bottom) obtained using LC–HRMS method 1. The ions labelled $[M+H-CH_3OH]^+$ and $[M+H+H_2O-CH_3OH]^+$ may result from in-source hydrolysis of methyl ketal **5** in the acidic mobile phase.

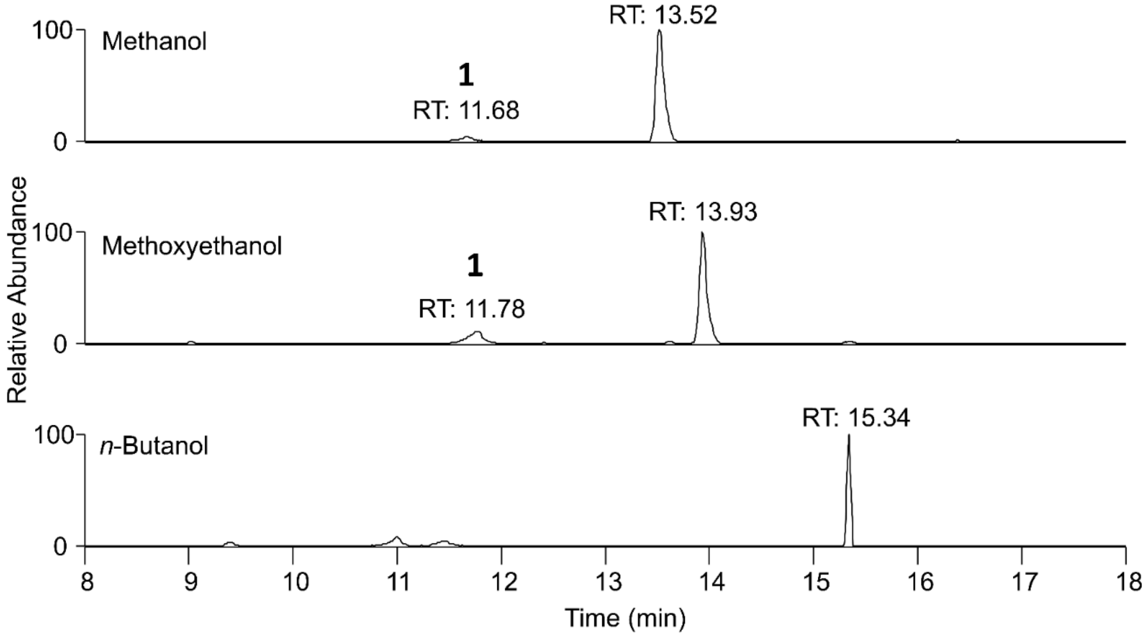

Figure S4. Extracted ion chromatograms from the formation of artefactual C-56 ketals of **1** by acid-catalyzed reaction with MeOH, methoxyethanol, and BuOH. Aliquots were injected into the LC–HRMS instrument immediately (~1 min) after dissolution of **1** in the respective alcohol.

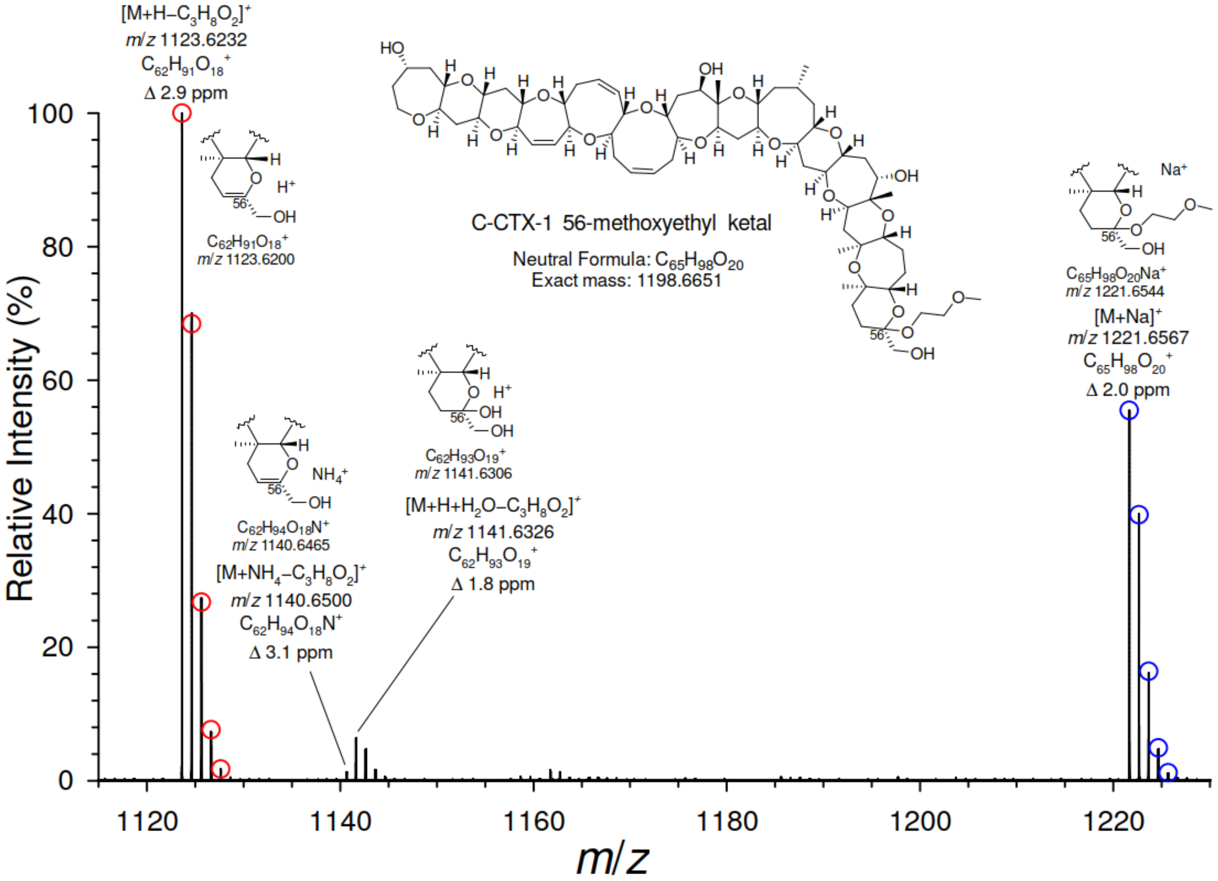

Figure S5. Full-scan LC–HRMS (method 1) spectrum (*m*/*z* 1115–1230) of C-CTX-1 56-methoxyethyl ketal in positive ionization mode (black line). Formulae assigned to the peaks at *m*/*z* 1123.6232 and 1221.6567 were the best fits obtained using the NRC Molecular Formula Calculator (v. Feb 2022; https://metrology.shinyapps.io/molecular-formula-calculator/) from the *m*/*z* and intensities of the five most abundant isotopomers, unrestricted numbers of C, O, and H atoms, up to one Na atom, and Senior rules. The circles show the relative isotopomer intensities predicted for the formulae with the Formula Calculator. The structure of the ketal, and plausible partial structures of the observed ions derived from it, together with their formulae and exact masses, are also shown. Each monoisotopic peak is labelled with the ion's assigned identity, accurate mass, assigned elemental composition, and mass error.

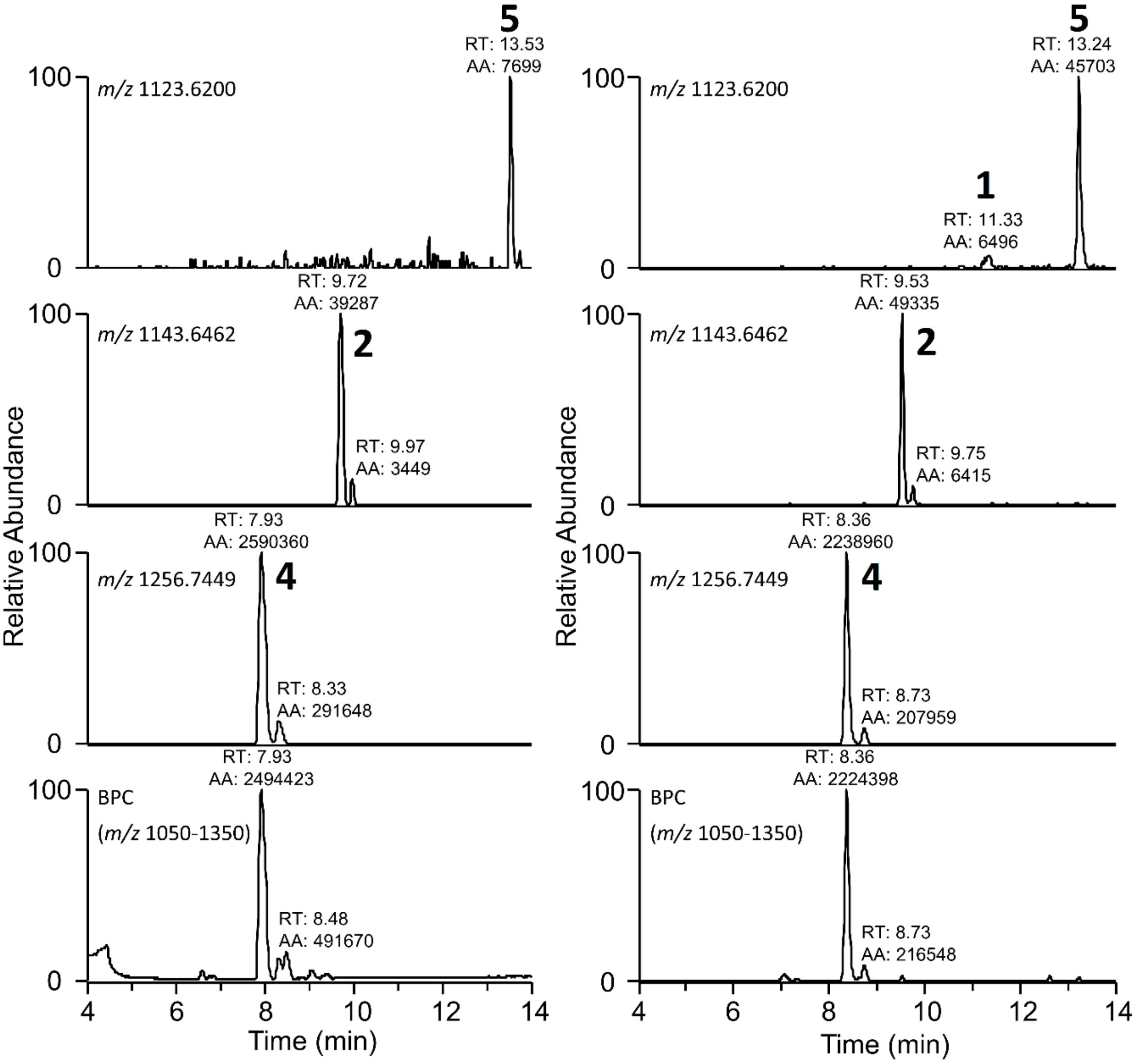

Figure S6. Extracted ion chromatograms from LC–HRMS analysis (LC–HRMS method 1) of the reductive amination of **1** with GRT in ciguatoxic sample-L in accordance with derivatization method 1A (on the left), and method 1B (on the right). Chromatograms are plotted using a mass tolerance of ± 5 ppm. The base-peak chromatogram (BPC, bottom) gives an impression of relative base peak abundances. The numbers in bold relate to the compound numbering in Figure 2.

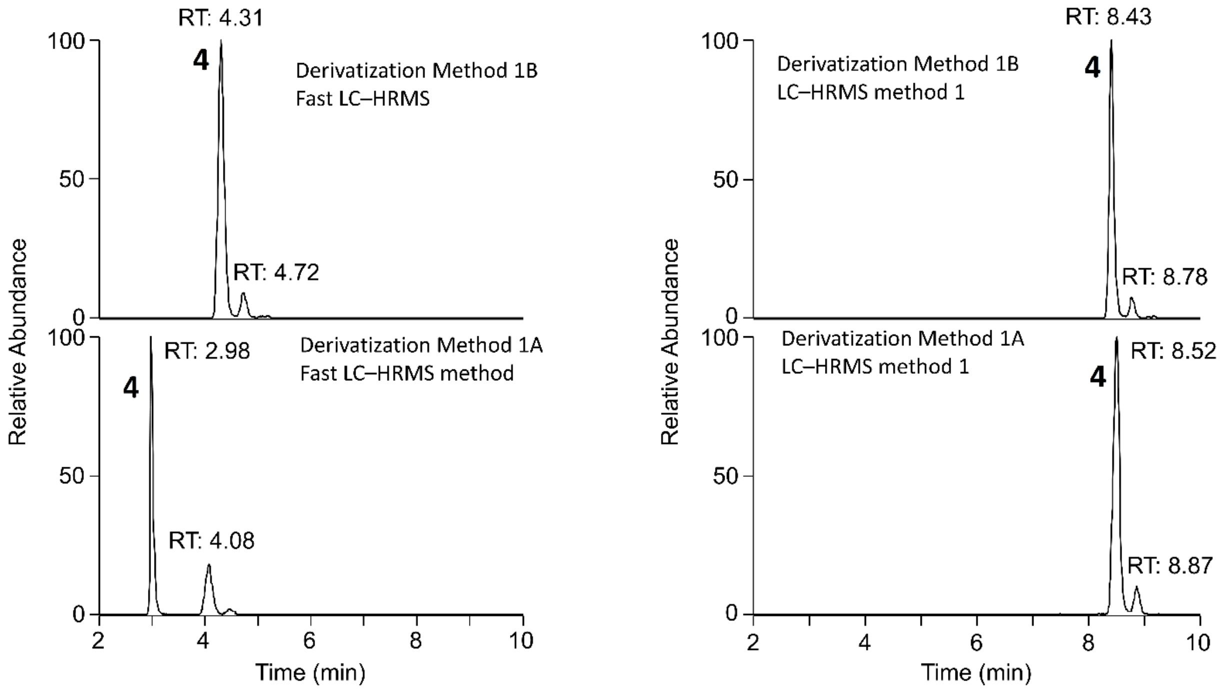

Figure S7. Extracted ion chromatograms (*m*/*z* 1256.7449, ± 5 ppm, M^+ of **4**) from LC–HRMS analysis using a modified, fast LC–HRMS method, and LC–HRMS method 1 on ciguatoxic sample-L in accordance with derivatization method 1A (on the bottom) and method 1B (on the top). The fast LC–HRMS method is a modification of LC–HRMS method 1 where the starting mobile phase composition has been changed from 20% of B to 30% B. The bottom-left trace shows an additional peak at 2.98 min, which is an artefact and the result of the presence of TFA in the sample.

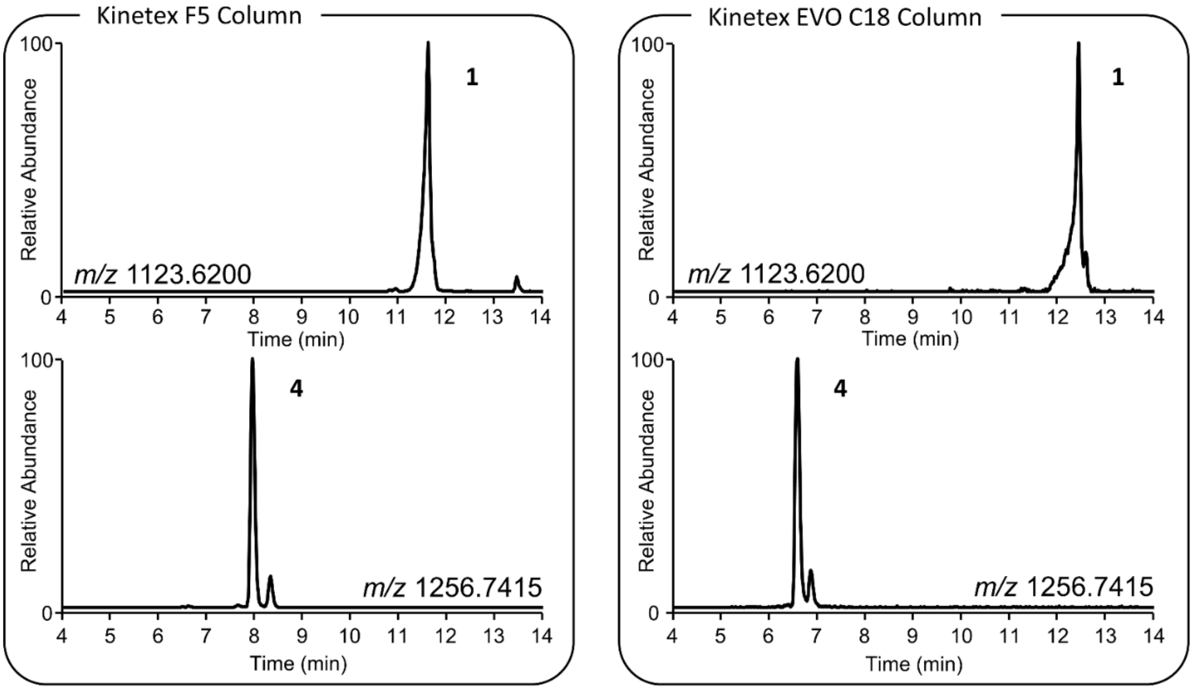

Figure S8. Extracted ion LC–HRMS chromatograms (± 5 ppm), obtained from a fish extract using LC–HRMS method 1 (left), or LC–HRMS method 2 (right) that used an octadecylsilane column, for C-CTX-1 (**1**) (top), and for C-CTX-1-GRT (**4**) (bottom) in sample-L.

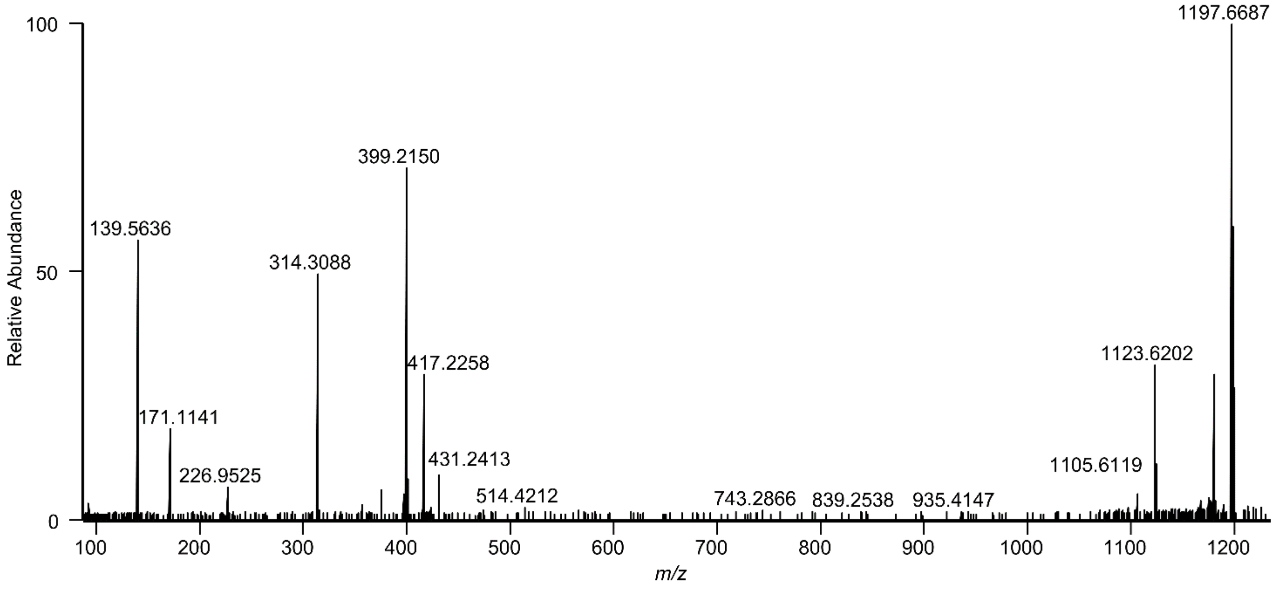

Figure S9. Part (*m*/*z* 87–1234) of the LC–HRMS/MS product ion spectrum from HCD of the M^+ ion of **4** (*m*/*z* 1256.7) applying a collision energy of 12 eV. The M^+ ion was the dominant ion, but is therefore outside the shown range. The product ion at *m*/*z* 1123.6202 is related to the loss of the GRT moiety.

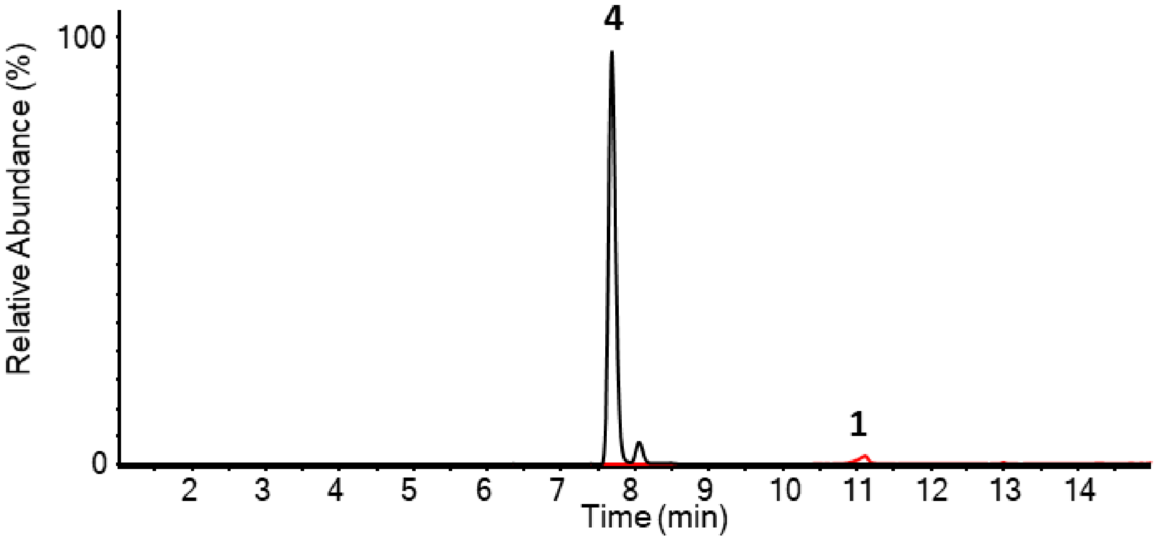

Figure S10. Overlaid LC–MS/MS chromatograms of sample D containing **1** (MRM transition: *m*/*z* 1123 → 1105; red) and the GRT-derivatized solution of sample D containing **4** (MRM transition: *m*/*z* 1256 → 60; black), both prepared at the same dilution.
